# Assessment of knowledge of drug-food interactions among healthcare professionals in public sector hospitals in eThekwini, KwaZulu-Natal

**DOI:** 10.1371/journal.pone.0259402

**Published:** 2021-11-03

**Authors:** Emmanuella Chinonso Osuala, Boikhutso Tlou, Elizabeth Bolanle Ojewole

**Affiliations:** 1 Discipline of Pharmaceutical Sciences, School of Health Sciences, College of Health Sciences, University of KwaZulu-Natal, Durban, South Africa; 2 Department of Public Health, School of Nursing & Public Health, College of Health Sciences, University of KwaZulu-Natal, Durban, South Africa; University of South Carolina College of Pharmacy, UNITED STATES

## Abstract

**Background:**

Foods and the nutrients they contain can interact with drugs and thereby interfere with their therapeutic safety and efficacy. Adequate knowledge of healthcare professionals (HCPs) about drug-food interactions can help in preventing potential drug-food interactions among patients. This study aimed to assess the knowledge of HCPs about common drug-food interactions.

**Methods:**

A cross-sectional study was carried out among 459 HCPs from three public hospitals in eThekwini district, KwaZulu-Natal between November 2018, and January 2019. Informed consent was obtained from the HCPs, and a structured questionnaire was thereafter administered. Data were analysed using SPSS® version 25. Factors associated with knowledge of the HCPs were determined using logistic regression analysis.

**Results:**

Of the 459 participants, 22.2% (n = 102) were doctors, 11.3% (n = 52) pharmacists, 63.8% (n = 293) nurses and 2.6% (n = 12) dietitians. Most of the HCPs were females 79.7% (n = 366), the mean age of the HCPs was 38.61±0.48. The knowledge score of the HCPs was 22.66±0.25 out of an overall score of 46. The HCPs poorly identified food types that interact with drugs and correct administration time of drugs relative to meals. Being a pharmacist (OR: 14.212, CI: 4.941–40.879, p<0.001), doctor (OR: 5.223, CI: 2.146–12.711, p<0.001), or a dietitian (OR: 5.476, CI: 1.103–27.191, p = 0.038) was associated with higher knowledge of drug-food interactions.

**Conclusion:**

The HCPs in this survey had low drug-food interaction knowledge. These findings suggest the need for additional training and educational courses for the HCPs on drug-food interactions.

## Introduction

Drugs and food both play a role in disease prevention and treatment. In many disease conditions, dietary interventions are part of the overall therapeutic strategy [1]. Patients need to consume proper food and nutrients, as well as safe and effective drugs [2]. However, the combination of medicines and food can also lead to undesirable interactions that can impact on therapeutic safety and efficacy [2]. Drug-food interaction is the result of a physical, chemical, or physiological relationship between a drug and food or nutrient [3]. Drug-food interactions may affect the pharmacokinetics (absorption, distribution, metabolism, and excretion) or pharmacodynamic properties of a drug and can cause decreased or increased bioavailability of the drug resulting in treatment failure or adverse events [4,5].

Furthermore, the influence of food on drugs depends on numerous variables, including physicochemical properties of the drug, as well as enzymes and transporters present in the gastrointestinal tract [6]. The presence and significance of drug-food interactions have been demonstrated by ample evidence [7]. For instance, warfarin has been reported to interact with vitamin K containing foods such as leafy green vegetables. Dietary vitamin K antagonizes the blood-thinning effect of warfarin, leading to unstable coagulation [8]. Angiotensin-converting enzyme inhibitors or potassium-sparing diuretics (e.g., spironolactone) increase potassium levels in the body; thus, excessive consumption of salt substitutes and potassium-containing foods like bananas and oranges can lead to hyperkalaemia [9]. Foods containing high dietary fiber have also been shown to interact with the absorption of drugs such as levothyroxine and digoxin [10,11]. Therefore, knowledge of drug-food interactions is essential to prevent these interactions.

The timing of food intake is an important factor that influences the occurrence of drug-food interactions. The presence of food may delay or reduce drug absorption, thus affecting the efficacy of such medicines [12,13]. For example, the absorption of the antidiabetic drug glipizide is decreased in the presence of food and it is recommended that glipizide is taken on an empty stomach, 30 minutes before food [14]. Thus, knowledge of the timing of food intake is also vital to achieve successful treatment and prevent adverse interactions.

Prevention of adverse interactions between food and drugs requires that healthcare professionals (HCPs) are knowledgeable [15,16]. HCPs need to provide patients with proper information on drug-food interactions, so they can recognize foods to avoid with their medications, as well as the right timing to take their medicines [16,17]. Thus, it is indispensable to ensure that HCPs’ knowledge regarding drug-food interaction is adequate. Previous studies have assessed HCPs’ knowledge of drug-food interactions, and several of these studies reported that the knowledge of the HCPs was inadequate [18–22]. However, in South Africa, data on the knowledge of HCPs as regarding drug-food interactions are lacking.

The purpose of this study was to assess knowledge of drug-food interactions among HCPs (doctors, pharmacists, nurses, and dietitians) working in three public hospitals in eThekwini, KwaZulu-Natal, South Africa. The information generated in this study would be instrumental in identifying areas where interventions are needed.

## Methods

### Study design and setting

This study was a descriptive cross-sectional study conducted to assess the knowledge of healthcare professionals (HCPs) about drug-food interactions. The study was carried out in three provincial hospitals in the eThekwini district, KwaZulu-Natal, Addington Hospital (ADH), King Edward VIII Hospital (KEH), and Prince Mshiyeni Memorial Hospital (PMMH). The hospitals have a bed count ranging from 571 to 1200 [23]. ADH and PMMH offer both district and regional healthcare services, and KEH offers tertiary care services to the whole of KwaZulu-Natal and part of the Eastern Cape [23]. The study was conducted among doctors, pharmacists, nurses, and dietitians between November 2018 and January 2019.

A total of 3,868 HCPs (518 doctors, 83 pharmacists, 3253 nurses, and 14 dietitians) formed the target population for this study. From the total number and proportions of the different HCPs, a minimum sample size of 364 was calculated using the Yamane formula with a 5% precision level and a 95% confidence level [24]. After allowing for a 30% non-response, a final aggregate sample size of 473 was obtained. Due to the low number of pharmacists and dietitians, all consenting pharmacists and dietitians working in the three hospitals were recruited to participate in the study. On-duty doctors and nurses were conveniently selected from the outpatient and the inpatient wards in the hospitals. Other HCPs such as physiotherapists, radiologists, and anaesthesiologists were excluded from this study because they are not actively involved in prescribing, dispensing or administering medications to patients.

### Data collection

An anonymous self-administered questionnaire was used to collect data from the consenting study participants. The questionnaire was adapted and modified from a previous tool used to test drug-food interaction knowledge of doctors in the published paper by Benni et al. [25]. Information on the demographics and knowledge of HCPs was obtained using the questionnaire. The demographic information collected were gender, ethnic group, age, level of education, occupation, years of work experience, and attendance of training on drug-food interactions. The knowledge section consisted of 25 dichotomous and multiple-choice questions. Questions 1–8 assessed the general knowledge of the HCPs about drug-food interactions, questions 9–18 measured the knowledge of interactions of specific foods and drugs, questions 19–23 measured knowledge of the timing of food intake relative to a drug, while questions 24 and 25 measured the knowledge of antihypertensive and antiretroviral drugs and food interactions.

The questionnaire was pre-tested for validity on 20 HCPs, after which minor adjustment was made to the tool before using it for the actual study. The average time taken to complete the questionnaire was 20 minutes. The data collected during the pre-test was not included in the main study analysis.

### Data analysis

The knowledge questions were grouped into four categories, and the overall knowledge score was computed by summing up the scores of each category with a total possible score of 46. For questions 1–8, each correct response was assigned a maximum score of 1 and incorrect response was scored 0. Questions 9–25 were multiple choice questions, and each question could have more than one correct response. Each correct response was given a score of 1 while incorrect responses were scored 0. The mean of the overall knowledge score (22.66) was used as the cut-off to classify the knowledge level as either low or high. HCPs who scored less than the mean knowledge score or within the exact mean cut-off were classified as having low knowledge, while those who scored above the mean were classified as having high drug-food interaction knowledge. The Statistical Package for the Social Sciences software (SPSS®) version 25 was used for data analysis. Continuous variables were expressed as mean ± standard error of the mean (SEM), while categorical variables were expressed as frequency and percentages. Chi-square was used to determine the relationship between the HCPs’ demographic characteristics and knowledge of drug-food interactions. Furthermore, binary logistic regression analysis was performed to determine the predictors of drug-food interaction knowledge. Statistical significance was set at a level of p ≤ 0.05.

### Ethical consideration

Ethical approval was obtained from the Biomedical Research Ethics Committee of the University of KwaZulu-Natal, South Africa (BE 371/18) and the KwaZulu-Natal department of health (KZ_201808_027). Gatekeeper permissions were also obtained from the three hospitals before starting data collection. The study participants were assured of their confidentiality and anonymity, and they voluntarily gave written informed consent.

## Results

### Demographic and general characteristics of the healthcare professionals

The total number of healthcare professionals (HCPs) in the survey was 459; of whom 22.2% (n = 102) were doctors, 11.3% (n = 52) pharmacists, 63.8% (n = 293) nurses and 2.6% (n = 12) dietitians. The overall response rate of HCPs was 97%. Most of the HCPs were females, 79.7% (n = 366) and 66.1% (n = 302) were Africans. The mean age of the HCPs was 38.61 ± 0.48, and the age category, 30–39 years, was the highest in frequency (n = 167, 37.6%). Less than half of the HCPs (n = 219, 47.7%) had over ten years of work experience, with 27% (n = 124) having between 5–9 years of work experience. Most of the HCPs 83.4% (n = 381) had not attended any training where they were informed about drug-food interactions. The summary of the demographic and general characteristics of the HCPs in this study is shown in Table 1.

**Table 1:** Demographic and general characteristics of the healthcare professionals.

Variable	Frequency (n)	Percentage (%)
**Gender (N = 459)**		
Male	93	20.3
Female	366	79.7
**Ethnic group (N = 457)**		
African	302	66.1
Indian/Asian	105	23.0
White	29	6.3
Coloured	21	4.6
**Age Category (N = 444)**		
29 years and below	91	20.5
30–39 years	167	37.6
40–49 years	100	22.5
50 years and above	86	19.4
**Education level (N = 459)**		
Certificate	55	12.0
Diploma	202	44.0
Bachelors	179	39.0
Postgraduate degree (Masters and postgraduate diploma)	23	5.0
**Occupation (N = 459)**		
Doctor	102	22.2
Pharmacist	52	11.3
Nurse	293	63.8
Dietitian	12	2.6
**Years of work experience (N = 459)**		
0–4 yrs.	116	25.3
5–9 yrs.	124	27.0
10–14 yrs.	106	23.1
15–19 yrs.	63	13.7
20 years and above	50	10.9
**Attended training on drug-food interactions (N = 457)**		
Yes	76	16.6
No	381	83.4

### Knowledge of healthcare professionals about drug-food interactions

The knowledge of the HCPs was assessed based on their responses to questions on drug-food interactions. The questions were grouped into four categories, as shown in Table 2, each of the categories had total scores of 8, 27, 5, and 6 respectively. The overall mean score (%) was calculated as the percentage of the overall mean score divided by the total score per category. The overall mean score of the HCPs was 22.66 ± 0.25 out of a total score of 46, which is equivalent to 49.3%. In general, the HCPs had low knowledge about drug-food interactions, as over half of the HCPs (56%) scored below the mean. The HCPs had high percentage scores in the questions on general knowledge (79.1%) and low percentage scores in the questions about interactions of specific food with drugs (39.0%) and timing of food intake relative to drugs (35.6%). Pharmacists had the highest scores across all question categories compared to the doctors, nurses, and dietitians (Table 2).

**Table 2:** Knowledge score of healthcare professionals about drug-food interactions (N = 459).

Knowledge scores per category of questions	Doctors mean score ± SEM	Pharmacists mean score ± SEM	Dietitians mean score ± SEM	Nurses mean score ± SEM	Overall mean score ± SEM	Overall mean score (%)
Healthcare professionals’ general knowledge regarding drug-food interactions (total score = 8)	6.92 ± 0.12	7.11 ± 0.10	7.04 ± 0.30	5.96 ± 0.09	6.33 ± 0.07	79.13
Knowledge of interaction of specific foods with drugs (total score = 27)	12.22 ± 0.39	13.50 ± 0.57	13.33 ± 1.38	9.32 ± 0.16	10.54 ± 0.17	39.04
Knowledge of timing of food intake relative to drugs (total score = 5)	2.11 ± 0.12	2.39 ± 0.15	1.75 ± 0.39	1.55 ± 0.07	1.78 ± 0.06	35.60
Knowledge of interaction of food with antihypertensive and antiretroviral drugs (total score = 6)	4.11 ± 0.13	4.54 ± 0.20	4.33 ± 0.41	3.95 ± 0.10	4.06 ± 0.07	67.67
Total score = 46	25.33 ± 0.54	27.49 ± 0.71	26.45 ± 1.70	20.72 ± 0.24	22.66 ± 0.25	49.26

Regarding questions on general knowledge about drug-food interactions, over 90% of the HCPs knew that some foods and drinks could interfere with the effectiveness of drugs in the body and that drugs can alter the nutritional status of a patient. Only 37.5% (n = 172) of the HCPs could identify that the elderly patients were at higher risk for drug-food interactions and 56.2% (n = 258) correctly identified all four levels (absorption, distribution, metabolism, and excretion) in which foods/beverages interact with drugs.

The HCPs were unable to identify many food types that can potentially interact with medications. For instance, few of the HCPs knew that a patient on theophylline should avoid consuming large quantities of tea (19.4%) and chocolates (21.6%), although a majority (85.6%) recognised that large amounts of coffee should be avoided. Also, a few of the HCPs (24.2%) knew that caffeine increases the toxicity of pseudoephedrine. Only 38% and 24% of HCPs correctly answered that taking tetracycline and fluoroquinolones with milk and iron-rich foods should be avoided. Most of the HCPs could not accurately identify the examples of foods that interact with monoamine oxidase inhibitors (MAOI), warfarin, and levothyroxine. The HCPs could not correctly identify cheese (70.4%), wine (59.9%), fermented products (61.9%) as possible to interact with MAOIs. Although the HCPs identified that spinach should be avoided in patients taking warfarin, they poorly identified other foods like broccoli (28.8%), green leafy lettuce (33.6%), and pork (25.5%). Most HCPs were unable to identify cabbage (63.8%), cauliflower (81.3%), and millet (67.5%) as foods that can interact with levothyroxine. About 40% of the HCPs correctly answered that alcohol should be avoided with metronidazole and antihistamines, while 64.7% knew that alcohol should be avoided with diazepam.

The HCPs had low knowledge about the timing of taking medications in relation to meals. Less than half identified the correct intake time of glipizide (32.9%), isoniazid (31.4%), nonsteroidal anti-inflammatory drugs (NSAIDs) (17.6%) and levothyroxine (30.7%) with a meal. While about 60% of the HCPs identified the correct intake time of omeprazole in relation to food.

### Factors associated with knowledge of the healthcare professionals

A significant association (p = 0.004) was obtained between gender and knowledge of drug-food interactions. However, knowledge of drug-food interactions was highly associated (p<0.001) with educational level and occupation, as shown in Table 3. There was no significant difference in the knowledge of healthcare professionals at the different levels of care.

**Table 3:** Associations between demographic characteristics and the healthcare professional’s knowledge of drug-food interactions.

Variable	Low Knowledge		High Knowledge		Total		p-value
	n	(%)	n	(%)	n	(%)	
**Gender (N = 459)**							
Male	40	43.0	53	57.0	93	100	0.004**
Female	218	59.6	148	40.4	366	100	
**Age Category (N = 444)**							
23–29 years	46	18.4	45	23.2	91	100	0.066
30–39 years	87	34.8	80	41.2	167	100	
40–49 years	67	26.8	33	17.0	100	100	
50 years and above	50	20.0	36	18.6	86	100	
**Education level (N = 459)**							
Certificate	37	67.3	18	32.7	55	100	<0.001***
Diploma	145	71.8	57	28.2	202	100	
Bachelors	73	40.8	106	59.2	179	100	
Postgraduate degree (Masters and postgraduate diploma)	3	13.0	20	87.0	23	100	
**Occupation (N = 459)**							
Doctor	37	36.3	65	63.7	102	100	<0.001***
Pharmacist	9	17.3	43	82.7	52	100	
Nurse	209	71.3	84	28.7	293	100	
Dietitian	3	25.0	9	75.0	12	100	
**Years of work experience (N = 459)**							
0–4 yrs.	65	56.0	51	44.0	116	100	0.973
5–9 yrs.	71	57.3	53	42.7	124	100	
10–14 yrs.	60	56.6	46	43.4	106	100	
15–19 yrs.	33	52.4	30	47.6	63	100	
20 years and above	29	58.0	21	42.0	50	100	
**Attended training on drug-food interactions (N = 457)**							
Yes	42	55.3	34	44.7	76	100	0.851
No	215	56.4	166	43.6	381	100	

**Significant association at p-value≤0.01

***Significant association at p-value≤0.001.

In the logistic regression analysis to predict factors associated with drug-food interaction knowledge of HCPs, only occupation remained statistically significant as shown in Table 4. Doctors (OR: 5.223, 95% CI: 2.146–12.711), pharmacists (OR: 14.212, 95% CI: 4.941–40.879), and dietitians (OR: 5.476, 95% CI: 1.103–27.191) were more likely to be knowledgeable as compared to nurses. HCPs with postgraduate degrees (OR: 2.728, 95% CI: 0.566–13.142) were likely to be more knowledgeable than those with certificates, but this was not significant.

**Table 4:** Binary logistic regression analysis of factors associated with knowledge of drug-food interactions (N = 457).

Variable	OR (95%CI)	p-value
**Gender**		
Female	-	-
Male	1.043 (0.594–1.832)	0.884
**Educational level**		
Certificate	-	-
Diploma	0.806 (0.424–1.532)	0.510
Bachelors	0.614 (0.235–1.604)	0.320
Postgraduate degree (Masters and postgraduate diploma)	2.728 (0.566–13.142)	0.211
**Occupation**		
Nurse	-	-
Doctor	5.223 (2.146–12.711)	<0.001***
Pharmacist	14.212 (4.941–40.879)	<0.001***
Dietitian	5.476 (1.103–27.191)	0.038*

CI- confidence interval

*Significant association at p-value≤0.05

***Significant association at p-value≤0.001.

## Discussion

This study assessed the knowledge of drug-food interactions among four groups of healthcare professionals (doctors, nurses, pharmacists, and dietitians) and determined the factors influencing their knowledge. To the best of our knowledge, this is the first study that assessed the knowledge of drug-food interactions among healthcare professionals (HCPs) in public hospitals in KwaZulu-Natal, South Africa. To interrogate the knowledge of these HCPs, a self-administered questionnaire was given to them, and scores were computed from the correctly answered questions. Overall, the HCPs had low knowledge of drug-food interactions, with a mean score of 22.66 ± 0.25 out of a maximum score of 46. It is possible that HCPs were not adequately trained on drug-food interactions, thereby giving rise to the low knowledge recorded. Our study findings are similar to those of previous studies in which doctors, nurses, and pharmacists were reported to have inadequate knowledge regarding drug-food interactions [18,20,21,26]. In our study, the knowledge score of the HCPs was slightly lower than those reported, possibly due to the types of questions that required the HCPs to identify specific food items that interact with drugs.

The HCPs in our study had high knowledge scores in two question categories (general knowledge, and knowledge of interaction of food with antihypertensive and antiretroviral drugs), while they obtained low knowledge scores in the others (knowledge of interaction of specific foods with drugs, and knowledge of timing of food intake relative to drugs). For the general knowledge questions, the majority of the HCPs were aware that food and drinks could interact with medications and that drugs can alter the nutritional status of a patient. Among the HCPs that had high knowledge, few knew that elderly patients (> 60 years) were at a greater risk of drug-food interactions. The elderly are at an increased risk of drug-food interactions due to altered physiological functions as a result of aging, multiple disease conditions, and polypharmacy [27]. Knowledge of the age group at risk for drug-food interactions is critical to ensure that they are prioritised for counselling as well as monitored for any potential interactions.

The HCPs exhibited low knowledge in identifying specific food items that can interact with drugs. Only a few of the HCPs could determine that dietary caffeine can interact with medicines such as theophylline and pseudoephedrine. Consuming caffeine while taking theophylline can lead to adverse effects such as headaches and nausea, and increased anxiety and nervousness with pseudoephedrine [13]. Similarly, few HCPs knew that milk and iron-rich food interact with antibiotics such as tetracycline and fluoroquinolones. Studies have shown that calcium and casein present in milk and dairy products can interact with fluoroquinolones, leading to decreased absorption and bioavailability of the medications [8]. The consumption of tyramine-containing foods, principally cheese, by patients on MAOIs was associated with severe hypertensive crisis, characterised by pulsating headache, palpitations, stiff neck, and nausea [28]. This interaction led to the development of strict dietary restrictions with MAOIs [29]. In our study, HCPs could not correctly identify food types to avoid when taking MAOIs. A possible reason for the low knowledge of the HCPs about the MAOI interaction could be because it is no longer commonly used as newer anti-depressants have replaced it.

Another significant finding of this study is the low knowledge of the HCPs about the correct administration time of medications relative to food. Comparable results were reported in a study in Palestine, where pharmacists lacked knowledge on the correct timing for drugs such as levothyroxine, NSAID, griseofulvin, and glipizide [18]. In our study, only about 30% of the HCPs identified that glipizide, isoniazid, and levothyroxine should be taken before food and 17.6% identified that NSAIDs should be taken with food. Taking medications with food or on an empty stomach can impact its systemic exposure. For instance, the bioavailability and maximum concentration of isoniazid are decreased in the presence of food intake which can impact on the therapeutic efficacy of the anti-tuberculosis medication [30]. Knowledge of the correct administration time of medicines is essential particularly among nurses as they administer medication to their patients. Therefore, poor knowledge of administration time can lead to medication error and compromise patient safety and effective therapy.

Knowledge of drug-food interactions was significantly associated with occupation in the logistic regression analysis. The pharmacists, doctors and dietitians were more likely to have high knowledge compared to the nurses. Overall, pharmacists had the highest knowledge score in all sections compared to the other HCPs. Similar findings were reported by Couris et al. (2000) in which the pharmacists had the highest overall knowledge score followed by the doctors and then the dietitians and nurses [31]. In our study, dietitians scored higher than the doctors in all sections except in the section about the timing of food intake relative to drug, however, the nurses had the lowest scores in all sections. Notably, the very small sample size of the dietitians and pharmacists may have had bearing on the findings; hence the results should be interpreted with caution. The better knowledge of the pharmacists underscores a possible role that pharmacists can play in educating other healthcare professionals about drug-food interaction. The rate of drug-food interactions was reduced in a hospital in Iran where a clinical pharmacist trained nurses about drug-food interactions [32].

Furthermore, although not significant, the HCPs with postgraduate degrees were three times more likely to be knowledgeable than those with other degrees (certificates). Having a postgraduate degree may expose you to advanced knowledge, hence, this advocates for a need for continued education among HCPs. Age of the HCPs, number of years of experience, and having attended training on drug-food interactions were not significantly associated with knowledge of the HCPs in our study. In this study, only a few of the HCPs had attended training on drug-food interactions possibly due to the low emphasis given to drug-food interactions in the clinical setting.

### Strengths

This study addresses the gap in the literature on knowledge of HCPs regarding drug-food interactions in the South African context. The study reveals particular areas where the HCPs have insufficient knowledge and demonstrates how the HCPs’ demographic and professional characteristics influence their knowledge of drug-food interactions. The study findings can be used as baseline information for designing interventions to improve the knowledge of the HCPs.

### Limitations

The study was limited to only one district in the KwaZulu-Natal province, and thus the study findings may not be generalizable to the whole of South Africa. The results of the dietitians and pharmacists need to be interpreted with caution, considering the very small sample, compared to the doctors and nurses.

### Recommendations

The findings from this study demonstrate the need for educating healthcare professionals to improve their knowledge of drug-food interactions. Education should also be provided to healthcare professionals in training to equip them with the necessary knowledge required in clinical practice. Comprehensive educational materials and resources should be designed for utilisation by healthcare professionals. These resources should be tailored to the South African context to improve their utility. Future studies assessing healthcare professionals’ knowledge of drug-food interactions should be carried out in different districts and provinces of South Africa and the private healthcare sector. The impact of training on the healthcare professional’s knowledge of drug-food interactions should also be investigated.

## Conclusion

Low knowledge of drug-food interactions was reported among the HCPs in this study. The HCPs had a high general knowledge of drug-food interactions but low knowledge on the specific food items that can interact with drugs and correct timing of food intake relative to drug. The pharmacists had higher knowledge scores across different question categories compared to the dietitians, doctors, and nurses, however, this should be interpreted with caution due to their small sample size. HCPs with a postgraduate degree were likely to have high knowledge of drug-food interactions. HCPs need to be conversant with common drug-food interactions to provide appropriate patient counselling and achieve better therapeutic outcomes. This study, therefore, highlights the need for educational interventions to improve the knowledge of DFIs, especially among nurses. Professional associations and educational institutions should emphasise teaching on drug-food interactions during the undergraduate training and organise educational courses for practicing HCPs.

## Supporting information

S1 Table Healthcare professional’s responses to the individual knowledge items. (DOCX)

S1 Questionnaire Healthcare professionals’ questionnaire (English). (DOCX)
