# Supplementary material for: Assessment of knowledge of drug-food interactions among healthcare professionals in public sector hospitals in eThekwini, KwaZulu-Natal
Source: PLoS One. 2021 Nov 3;16(11):e0259402. doi: 10.1371/journal.pone.0259402 (PMC8565720; doi:10.1371/journal.pone.0259402)
Supplement: S1 Table — (DOCX) [file pone.0259402.s001.docx]

**Supporting Information**

**S1 Table. Healthcare professional’s responses to the individual knowledge items**

| **Knowledge questions** | | **Frequency of correct answer**  **n (%)** | **Frequency of wrong answer**  **n (%)** | **Missing response**  **n (%)** |
| --- | --- | --- | --- | --- |
| **Questions 1-8: Healthcare professionals’ general knowledge regarding drug-food interactions** | | | | |
| **K1** | Some foods can interfere with the effectiveness of drugs in the body | 421 (91.7) | 32 (7.0) | 6 (1.3) |
| **K2** | Some drinks can interfere with the effectiveness of drugs in the body | 438 (95.4) | 15 (3.3) | 6 (1.3) |
| **K3** | Some foods can increase or decrease the action of a drug | 418 (91.1) | 35 (7.6) | 6 (1.3) |
| **K4** | Some drugs can alter the nutritional status of a patient | 414 (90.2) | 40 (8.7) | 5 (1.1) |
| **K5** | Age group of patients at greater risk for DFI | 172 (37.5) | 254 (55.3) | 33 (7.2) |
| **K6** | Factors that influence DFI | 339 (73.9) | 108 (23.5) | 12 (2.6) |
| **K7** | DFI can occur when drugs interact with diet, iron/vitamin supplements, alcohol, and fruit juices | 314 (68.4) | 137 (29.8) | 8 (1.7) |
| **K8** | Mechanism of interaction of food/beverages with drugs | 258 (56.2) | 194 (42.3) | 7 (1.5) |
| **Questions 9-17: Knowledge of HCPs about individual interactions of food with drugs** | | | | |
|  | **Questions** | **Correct answer**  **n (%)** | **Unanswered**  **n (%)** | **Wrong**  **answer**  **n (%)** |
| **K9A** | A patient on Theophylline should avoid consuming large quantities of a) Tea | 89 (19.4) | 370 (80.6) | - |
| **K9B** | A patient on Theophylline should avoid consuming large quantities of b) Coffee | 393 (85.6) | 66 (14.4) | - |
| **K9C** | A patient on Theophylline should avoid consuming large quantities of c) Chocolates | 99 (21.6) | 360 (78.4) | - |
| **K10A** | A patient taking Antibiotics such as tetracycline and fluoroquinolones should avoid? a) Milk | 172 (37.5) | 287 (62.5) | - |
| **K10B** | A patient taking Antibiotics such as tetracycline and fluoroquinolones should avoid? b) Dairy products | 244 (53.2) | 215 (46.8) | - |
| **K10C** | A patient taking Antibiotics such as tetracycline and fluoroquinolones should avoid? c) Iron-rich food | 108 (23.5) | 351 (76.5) | - |
| **K10D** | A patient taking Antibiotics such as tetracycline and fluoroquinolones should avoid? d) Meat | - | 418 (91.1) | 41 (8.9) |
| **K11A** | A patient on Monoamine oxidase inhibitors should avoid a) Cheese | 136 (29.6) | 323 (70.4) | - |
| **K11B** | A patient on Monoamine oxidase inhibitors should avoid b) Fresh meats | - | 412 (89.8) | 47 (10.2) |
| **K11C** | A patient on Monoamine oxidase inhibitors should avoid c) wine | 184 (40.1) | 275 (59.9) | - |
| **K11D** | A patient on Monoamine oxidase inhibitors should avoid d) Fava beans | 46 (10.0) | 413 (90.0) | - |
| **K11E** | A patient on Monoamine oxidase inhibitors should avoid e) Fermented products | 175 (38.1) | 284 (61.9) | - |
| **K12A** | A patient taking antibiotics should avoid acidic foods such as a) Tomato sauce | 188 (41.0) | 271 (59.0) | - |
| **K12B** | A patient taking antibiotics should avoid acidic foods such as b) Potato | - | 449 (97.8) | 10 (2.2) |
| **K12C** | A patient taking antibiotics should avoid acidic foods such as c) Coffee | 91 (19.8) | 368 (80.2) | - |
| **K12D** | A patient taking antibiotics should avoid acidic foods such as d) Citrus juices | 301 (65.6) | 158 (34.4) | - |
| **K13A** | Caffeine increases the risk of toxicity of the following drugs a) Pseudoephedrine | 111 (24.2) | 348 (75.2) | - |
| **K13B** | Caffeine increases the risk of toxicity of the following drugs b) Theophylline | 370 (80.6) | 89 (19.4) | - |
| **K14A** | The following drugs should be taken with a low-fat diet a) Griseofulvin | - | 324 (70.6) | 135 (29.4) |
| **K14B** | The following drugs should be taken with a low-fat diet b) Albendazole | - | 319 (69.5) | 140 (30.5) |
| **K14C** | The following drugs should be taken with a low-fat diet c) Esomeprazole | 196 (42.7) | 263 (57.3) | - |
| **K15A** | A patient should avoid alcohol with metronidazole | 182 (39.7) | 277 (60.3) | - |
| **K15B** | A patient should avoid alcohol with diazepam | 297 (64.7) | 162 (35.3) | - |
| **K15C** | A patient should avoid alcohol with antihistamines | 164 (35.7) | 295 (64.3) | - |
| **K16A** | A patient on warfarin should avoid spinach | 242 (52.7) | 217 (47.3) | - |
| **K16B** | A patient on warfarin should avoid broccoli | 132 (28.8) | 327 (71.2) | - |
| **K16C** | A patient on warfarin should avoid green leaf lettuce | 154 (33.6) | 305 (66.4) | - |
| **K16D** | A patient on warfarin should avoid pork | 117 (25.5) | 342 (74.5) | - |
| **K16E** | A patient on warfarin should avoid mushroom | - | 428 (93.2) | 31 (6.8) |
| **K17A** | A patient on levothyroxine must avoid cabbage | 166 (36.2) | 293 (63.8) | - |
| **K17B** | A patient on levothyroxine must avoid lean meat | - | 373 (81.3) | 86 (18.7) |
| **K17C** | A patient on levothyroxine must avoid cauliflower | 86 (18.7) | 373 (81.3) | - |
| **K17D** | A patient on levothyroxine must avoid millet | 149 (32.5) | 310 (67.5) | - |
| **K18** | Long period of consumption of garlic/ginger along with warfarin should be avoided | 247 (53.8) | 191 (41.6) | - |
| **Questions 19-23: Knowledge of HCPs about timing of food intake relative to drugs** | | | | |
|  | **Questions** | **Correct**  **n (%)** | **Incorrect**  **n (%)** | **Missing response**  **n (%)** |
| **K19** | Omeprazole should be taken before food | 276 (60.1) | 163 (35.5) | 20 (4.4) |
| **K20** | Glipizide should be taken before food | 151 (32.9) | 265 (57.7) | 43 (9.4) |
| **K21** | Isoniazid should be taken before food | 144 (31.4) | 283 (61.7) | 32 (6.9) |
| **K22** | NSAIDs should be taken with food | 81 (17.6) | 349 (76.0) | 29 (6.3) |
| **K23** | Levothyroxine should be taken before food | 141 (30.7) | 272 (59.3) | 46 (10.0) |
| **Questions 24-25: Knowledge of interaction of food with antihypertensive and antiretroviral drugs** | | | | |
|  | **Questions** | **Correctly answered**  **n (%)** | **Unanswered**  **n (%)** |  |
| **K24A** | Propranolol/ACEi must be taken on empty stomach | 20 (4.4) | 439 (95.6) |  |
| **K24B** | Spironolactone must be avoided with potassium rich foods | 66 (14.4) | 393 (85.6) |  |
| **K24C** | Hypertensive patients require low salt diet | 242 (52.7) | 217 (47.3) |  |
| **K24D** | All of the above | 216 (47.1) | 243 (52.9) |  |
| **K25A** | Lopinavir/Ritonavir must be taken with food | 123 (26.8) | 336 (73.2) |  |
| **K25B** | Didanosine and Indinavir must be taken on empty stomach | 31 (6.8) | 428 (93.2) |  |
| **K25C** | Zidovudine can be taken without relation to food intake | 65 (14.2) | 394 (85.8) |  |
| **K25D** | All of the above | 248 (54.0) | 211 (46.0) |  |
