# Supplementary material for: Assessment of knowledge of drug-food interactions among healthcare professionals in public sector hospitals in eThekwini, KwaZulu-Natal
Source: PLoS One. 2021 Nov 3;16(11):e0259402. doi: 10.1371/journal.pone.0259402 (PMC8565720; doi:10.1371/journal.pone.0259402)
Supplement: S1 Questionnaire — (DOCX) [file pone.0259402.s002.docx]

**S2 Questionnaire. Healthcare professionals’ questionnaire (English)**

SECTION 1: SOCIO DEMOGRAPHICS

Please indicate the following using a tick ():

1. Gender

Male ⧠

Female ⧠

2. Ethnic group

African ⧠

Indian ⧠

White ⧠

Coloured ⧠

Others, please specify __________________________________

3. Age (Please state): ______________years old

4. Level of education obtained:

Diploma ⧠

Bachelor’s degree ⧠

Master’s degree ⧠

Others (please specify) ___________________

5. Occupation:

Doctor ⧠

Pharmacist ⧠

Nurse ⧠

Dietitian ⧠

6. Years of work experience

0-4 years ⧠ 5-9 years ⧠ 10-14 years ⧠ 15-19 years ⧠ ≥20 years ⧠

7. Have you attended any training where you were informed about drug-food interactions?

Yes ⧠ No ⧠

SECTION 2: KNOWLEDGE REGARDING DRUG-FOOD INTERACTIONS

|  | Questions 1- 4, Please tick either Yes or No | YES | NO |
| --- | --- | --- | --- |
| 1 | Some foods can interfere with the effectiveness of drugs in the body? |  |  |
| 2 | Some drinks can interfere with the effectiveness of drugs in the body? |  |  |
| 3 | Some foods can increase or decrease the action of a drug? |  |  |
| 4 | Some drugs can alter the nutritional status of a patient? |  |  |

Questions 5 – 8, Please TICK the most correct answer:

5. The following age group of patients is at a greater risk for drug-food interactions?

a) Less than 1 yr.

b) 1-4 yrs

c) 5-14 yrs

d)15-45 yrs

e) 46-59 yrs

f) Greater than 60 yrs.

6. Drug-food interactions are influenced by

a) Person’s age ⧠

b) An individual’s health status ⧠

c) The dosages (tablets, capsules) of medication ⧠

d) Taking many medications at the same time ⧠

e) All of the above ⧠

7. Drug-food interactions can occur when drugs interact with the following

a) Diet ⧠

b) Iron/ Vitamin supplements ⧠

c) Alcohol and fruit juices ⧠

d) All of the above ⧠

8. At what level do the food/ beverages interact with the drugs?

a) Absorption ⧠

b) Distribution ⧠

c) Metabolism ⧠

d) Excretion ⧠

e) All of the above ⧠

Questions 9 - 17, Please TICK all applicable answers:

9. A patient on Theophylline should avoid consuming large quantities of

a) Tea ⧠

b) Coffee ⧠

c) Chocolates ⧠

10. A patient taking Antibiotics such as tetracycline and fluoroquinolones should avoid?

a) Milk ⧠

b) Dairy products ⧠

c) Iron-rich food ⧠

d) Meat ⧠

11. A patient on Monoamine oxidase inhibitors should avoid

a) Cheese ⧠

b) Fresh meats ⧠

c) Wine ⧠

d) Fava beans ⧠

e) Fermented products ⧠

12. A patient taking antibiotics should avoid acidic foods such as

a) Tomato sauce ⧠

b) Potato ⧠

c) Coffee ⧠

d) Citrus juices ⧠

13. Caffeine increases the risk of toxicity of the following drugs

a) Pseudoephedrine ⧠

b) Theophylline ⧠

14. The following drugs should be taken with a low-fat diet

a) Griseofulvin ⧠

b) Albendazole ⧠

c) Esomeprazole ⧠

15. A patient should avoid alcohol with the following drugs

a) Metronidazole ⧠

b) Diazepam ⧠

c) Antihistamines ⧠

16. A patient on Warfarin should avoid these foods

a) Spinach ⧠

b) Broccoli ⧠

c) Green leaf lettuce ⧠

d) Pork ⧠

e) Mushroom ⧠

17. Patients on Levothyroxine for hypothyroidism must avoid foods like

a) Cabbage ⧠

b) Lean meat ⧠

c) Cauliflower ⧠

d) Millet ⧠

18. Long period of consumption of garlic/ginger along with warfarin should be avoided?

Yes ⧠

No ⧠

Questions 19 - 23, circle the correct option. With relation to timing of food intake,

19. Omeprazole should be taken (before / with / after)

20. Glipizide should be taken (before / with / after)

21. Isoniazid should be taken (before / with / after)

22. NSAIDs should be taken (before / with / after)

23. Levothyroxine (before / with / after)

Questions 24 - 25, please TICK the correct answer:

24. Food and Antihypertensive drugs

a) Propranolol, ACE Inhibitors must be taken on empty stomach.

b) Spironolactone must be avoided with potassium rich foods.

c) Hypertensive patients require low salt diet.

d) All of the above

25. Food and Anti-Retroviral therapy (ART) drugs

a) Lopinavir / Ritonavir must be taken with food

b) Didanosine and Indinavir must be taken on empty stomach

c) Zidovudine can be taken without relation to food intake.

d) All of the above
